# Treatment of Severe Community-Acquired Pneumonia with Oral Amoxicillin in Under-Five Children in Developing Country: A Systematic Review

**DOI:** 10.1371/journal.pone.0066232

**Published:** 2013-06-25

**Authors:** Rashmi Ranjan Das, Meenu Singh

**Affiliations:** 1 Department of Pediatrics, All India Institute of Medical Sciences (AIIMS), Bhubaneswar, India; 2 Department of Pediatrics, Post-Graduate Institute of Medical Education and Research (PGIMER), Chandigarh, India; Beijing Institute of Microbiology and Epidemiology, China

## Abstract

**Objective:**

To assess the evidence regarding efficacy of oral amoxicillin compared to standard treatment for WHO-defined severe community acquired pneumonia in under-five children in developing country.

**Design:**

Systematic review and meta-analysis of data from published Randomized trials (RCTs).

**Data sources:**

MEDLINE (1970– July 2012) via PubMed, Cochrane Central Register of Controlled Trials (CENTRAL, The Cochrane Library, Issue 7, July 2012), and EMBASE (1988– June 2012).

**Methods:**

Eligible trials compared oral amoxicillin administered in ambulatory setting versus standard treatment for WHO-defined severe community acquired pneumonia in children under-five. Primary outcomes were proportion of children developing treatment failure at 48 hr, and day 6. GRADE criteria was used to rate the quality of evidence.

**Results:**

Out of 281 full text articles assessed for eligibility, 5 trials including 12364 children were included in the meta-analysis. Oral amoxicillin administered either in hospital or community setting is effective in treatment of severe pneumonia and is not inferior to the standard treatment. None of the clinical predictors of treatment failure by 48 hr (very severe disease, fever and lower chest indrawing, and voluntary with-drawl and loss to follow up) was significant between the two groups. The clinical predictors of treatment failure that were significant by day 6 were very severe disease, inability to drink, change of antibiotic, and fever alone. The effect was almost consistent across the studies.

**Conclusion:**

Though oral amoxicillin is effective in treatment of severe CAP in under-five children in developing country, the evidence generated is of low-quality. More trials with uniform comparators are needed in order to strengthen the evidence.

## Background

Worldwide, pneumonia is a leading cause of morbidity and mortality in children less than 5 years old. About 19% of deaths in such children are attributable to pneumonia (2 million deaths per year) [1], [2]. Two-thirds of these deaths happen during infancy, and more than 90% are in developing countries**.** One explanation for this higher mortality associated with pneumonia in developing countries is the high prevalence of a bacterial etiology (in up to 74% in some studies) [3]. The increase in antibiotic resistance, inadequate access to healthcare facilities, inability to implement vaccines against *Haemophilus influenzae* type-b and *Streptococcus pneumoniae* on a large scale in most parts of the developing world are the important hurdles for reducing mortality from pneumonia.

Standard guidelines developed by WHO recommend that children with non-severe pneumonia be treated at home with oral antibiotics, and those with severe pneumonia be admitted and given parenteral antibiotics (benzylpenicillin or ampicillin) [4]. Application of these guidelines in developing countries has lead to a decrease in mortality from acute respiratory infection [5]. In a developing or low income country, there are several drawbacks of admission to a health care facility for administration of parenteral antibiotics. These include, short supply or periodically unavailability of administration equipments preventing delivery of recommended treatment, increased cost of care, and poor transport facility or inaccessibility to hospital in some cases. In addition, children with severe pneumonia are prone for contracting nosocomial infections as a result of weak immunity and crowded hospital wards. Trials from developing countries have shown that, oral amoxicillin might be a possible alternative to standard treatment in children with a clinical diagnosis of WHO defined severe pneumonia [6]–[12]. If oral amoxicillin is proved to be as effective as injectable antibiotics in the treatment of severe pneumonia, then substantial improvements in access to appropriate care, nosocomial complications, iatrogenic infections, and costs could be achieved with its widespread use. A Cochrane review updated in 2006 concluded that, oral antibiotics appear to be as effective as parenteral antibiotics in the treatment of severe pneumonia in children [13]. Though additional trials have been published after 2006, no published systematic review has specifically looked for the evidence behind use oral amoxicillin in the treatment of severe community acquired pneumonia in children. So, the purpose of this systematic review would be to examine the available evidence regarding the efficacy of administering oral amoxicillin compared to either injectable antibiotics or cotrimoxazole and then referral to hospital, in the treatment of WHO-defined severe community acquired pneumonia in children aged 2–59 months in developing countries.

## Methods

### Types of Studies

Randomized clinical trials (including cluster trials) were included.

### Types of Participants

Children of 2–59 months of age with a clinical diagnosis of WHO-defined severe pneumonia were included. Severe pneumonia was defined as cough or difficulty breathing along with lower chest indrawing. Those with very severe disease (as defined by WHO; inability to drink, abnormal sleepiness, cyanosis, convulsions, and severe malnutrition), radiological consolidation or effusion, immunodeficiency including HIV, and severe malnutrition were excluded.

### Types of Interventions

The intervention group was treated with daily oral amoxicillin. The control group was treated either with injectable antibiotics followed by oral amoxicillin at home or was given first dose of cotrimoxazole and then referred to hospital. We also compared administration of amoxicillin at home versus at hospital. Any dosage schedule of amoxicillin was considered.

### Types of Outcome Measures

Trials measuring the following outcomes were included in the review.

#### Primary outcome measures

1. Proportion of children developing treatment failure at 48 hr, and day 6.

#### Secondary outcome measures

1. Proportion of children developing treatment failure or relapse by day 14.
2. Proportion of children developing treatment failure or relapse between day 6 and 14.
3. Clinical predictors of “failure of oral treatment” by 48 hr, day 6, and day 14.
4. Risk of death

For primary outcome, treatment failure is defined as appearance of any signs of very severe pneumonia, hospitalization, change of antibiotic, fever greater than 38°C, loss to follow up or voluntary withdrawal of consent, serious adverse event considered related to amoxicillin, or death.

For secondary outcome, treatment failure or relapse is defined as restart of any antibiotic due to reappearance of any of the danger signs, lower chest indrawing or fast breathing, fever greater than 38°C, left against medical advice (if admitted) or voluntary withdrawal of consent.

Hospitalization rate was defined as need for hospitalisation in children who were getting treatment on an ambulatory (outpatient) basis.

### Search Methodology

We searched the Cochrane Central Register of Controlled Trials (CENTRAL) (*The Cochrane Library*, Issue 7), which contains the Cochrane Acute Respiratory Infection (ARI) Group and the Cochrane Infectious Diseases Group Specialized Registers; MEDLINE (1970 to July 2012); EMBASE (1988 to June 2012). For further identification of ongoing trials, the website www.clinicaltrials.gov was searched and relevant trials were screened for eligibility of inclusion in the review. For MEDLINE search, following search terms were adopted: ((exp Pneumonia/) OR pneumonia OR lower respiratory tract infection$ OR LRTI OR lower respiratory infection$) AND (exp Antibacterial agents/OR antibiotics) AND (exp Child/OR child OR children exp Infant/OR infant OR infants OR paediatric OR pediatric) OR Child, Preschool/OR toddler OR preschool*. Two independent reviewers reviewed the search results to identify relevant original human clinical or field trials. Studies that focused on the effects of oral amoxicillin for the treatment of non-severe pneumonia or other respiratory infections were excluded from the analysis. Additional studies were identified through manual searches of reference lists of the originally identified studies as well as reviews on the subject. No language restrictions were applied.

### Data Extraction

Data extraction was done using a data extraction form that was designed and pilot tested *a priori*. The two authors (RRD, MS) independently extracted data from included studies, including year, setting (country, type of population, socioeconomic status, baseline under-five mortality, mortality due to pneumonia, etiological agents for pneumonia), exposure/intervention (oral amoxicillin administered at home or hospital, injectable antibiotics, other oral antibiotics used), results (outcome measures, effect, significance), and sources of funding/support. Disagreements in extracted data were resolved through discussion.

### Assessment of Risk of Bias in Included Studies

Two review authors (RRD, MS) independently assessed the methodological quality of the selected trials by using methodological quality assessment forms. We undertook quality assessment of the trials using the criteria outlined in the *Cochrane Handbook for Systematic Reviews of Interventions* [14]. Any disagreements between the two review authors were resolved through discussion.

### Data Analysis

The data from various studies were pooled and expressed as standardized mean difference (SMD) with 95% confidence interval (CI). P-value <0.05 was considered significant. Assessment of heterogeneity was done by I-squared (I^2^) statistics. If there was a high level of heterogeneity (>50%), we tried to explore this by subgroup analysis. A fixed effects model was initially conducted. If significant heterogeneity existed between trials, potential sources of heterogeneity were considered and where appropriate a random effects model was used. RevMan (Review Manager) version 5 was used for all the analyses [15].

## Results

### Description of Studies

**Figure 1:**
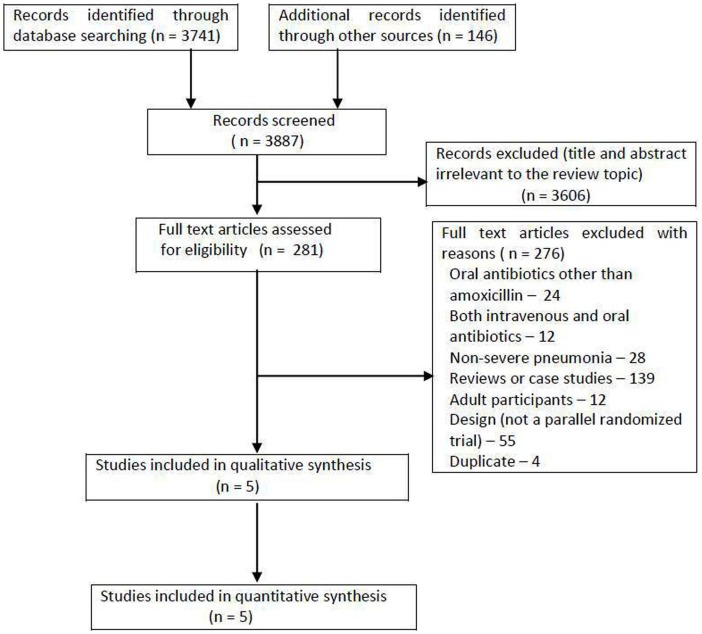
Flow diagram of search results.

Out of 3887 initial hits, 281 full text articles were assessed for eligibility, of which 276 were excluded (**Figure 1**). A total of 5 trials including 12364 children (intervention = 6585, control = 5779) were considered as potentially eligible for inclusion in the meta-analysis (**Table 1**) [6]–[10]. All the included trials were conducted in developing countries, with majority being conducted in Pakistan. So, the characteristics of study population, as well as etiological profile of pneumonia, are supposed to be more uniform in the included studies. All trials used WHO definitions of severe pneumonia. Two trials were purely community based involving lady health workers [8], [10] where as in other three trials [6], [7], [9] children were hospitalized for initial 48 hrs. In two trials [6], [7] amoxicillin compared with injectable penicillin or ampicillin, and in another trial, control group received oral cotrimoxazole followed by referral [8]. The dose of amoxicillin ranged from 45 to 90 mg/kg/day in different trials. Children <2 months (including neonates) were not included.

**Table 1:** Characteristics of included studies.

Study	Setting	Participants	Intervention	Outcomes measured	Treatment failure	Comments
Addo-Yobo et al [6] (non-blind, equivalence study)	Paediatric departments of tertiary care facilities at 9 international sites. Recruitment period: May 1999 to May 2002	Children aged 3–59 months with WHO-defined severe pneumonia. Oral amoxicillin (n = 857) or parenteral penicillin (n = 845)	**Intervention arm:** admitted for 48 hr and received oral amoxicillin (45 mg/kg/day), if improved discharged with a 5-day course of oral amoxicillin.**Control arm:** admitted for 48 hr and received parenteral penicillin (200000 IU/kg/day), if improved discharged with a 5-day course of oral amoxicillin, and if treatment failure then treated with a local alternative standard therapy.	**Primary outcome:** treatment failure up to or at first 48 hr.**Secondary outcome:** treatment failure at 5 or 14 day follow-up visits.	Any of the following: appearance of danger signs, low oxygen saturation, persistence of lower chest indrawing at 48 hr, life-threatening or serious adverse drug reaction, received another antibiotic, newly diagnosed co-morbid condition, parents or guardian withdrew consent, child left against medical advice, death.	Treatment failure rate was 19% in each arm at 48 hr. 3% of children <12 months, and 6% of >12 months age progressed to very severe disease on oral amoxicillin. Microbiological diagnosis was done. Serious adverse events reported (amoxicillin group –8, penicillin group –22) were: death (12), rash (5), diarrhoea (5), penicillin allergy (2), anaemia and malaria (one), severe malaria (3), and unspecified events (2).
Hazir et al [7](non-blind, equivalence study)	Paediatric departments of tertiary care facilities at 7 sites in Pakistan. Recruitment period: February 2005 to August 2006	Children aged 3–59 months with WHO-defined severe pneumonia. Oral amoxicillin (n = 1025) or parenteral ampicillin (n = 1012)	**Hospitalized group:** admitted for 48 hr and received parenteral ampicillin (100 mg/kg/day) followed by 3 days oral amoxicillin (80–90 mg/kg/day),**Ambulatory group:** treatment with 5 days oral amoxicillin (80–90 mg/kg/day).	**Primary outcome:** treatment failure up to or on day 6.**Secondary outcome:** treatment failure between day 6 or and day 14; relapse.	Any of the following: clinical deterioration, inability to take oral medication, co-morbid condition requiring antibiotic, persistence of fever >38°C with LCI from day 3 to 6, either fever or LCI alone at day 6, hospitalization related to pneumonia, serious adverse event, LAMA or lost to follow-up, voluntary withdrawal of consent, death.	Treatment failure in hospital and ambulatory arms was 5.8% vs 3.5% at 48 hr and 8.6% vs 7.5% by day 6. Microbiological diagnosis was not done. Five children died (ambulatory group –1, hospitalized group –4). None of the deaths were considered to be associated with study treatment, and there were no serious adverse events reported in the trial.
Bari et al [8] (cluster randomized)	Haripur district in Pakistan, community based involving lady health workers. Recruitment period: April 2008 to December 2009	Children aged 2–59 months with WHO-defined severe pneumonia. Oral amoxicillin (n = 1857) or Co-trimoxazole (n = 1354) followed by referral.	**Intervention clusters:** oral amoxicillin for 5 days (80–90 mg/kg/day or 375 mg BD for 2–11 months and 625 mg BD for those 12–59 months).**Control clusters:** first dose of oral co-trimoxazole (2–11 months, SMZ 200 mg plus TMP 40 mg; 12 months - 5 yrs, SMZ 300 mg plus TMP 60 mg) and then referred for standard care.	**Primary outcome:** development of clinical treatment failure by day 6.**Secondary outcome:** clinical relapse on days 7–14.	Appearance of a danger sign (unable to drink or breastfeed, convulsions, vomiting after ingestion of food or drink, and abnormally sleepy or difficult to wake), temperature at least 100°F and LCI on day 3, fever or LCI alone on day 6, and change of antibiotic (through self-referral or by carers).	Treatment failure in intervention and control cluster was, 9% and 18% by day 6. Microbiological diagnosis was not done. Three deaths occurred, one of which was in the intervention group. Reported adverse events were diarrhoea (n = 4) and skin rash (n = 1) in the intervention clusters and diarrhoea (n = 3) in the control clusters.
Patel et al [9] (non-blind, equivalence study)	Paediatric departments of tertiary care facilities of 6 centres in India. Recruitment period: October 2008 to March 2011	Children aged 3–59 months with WHO-defined severe pneumonia. Oral amoxicillin at home (n = 505) or oral amoxicillin at hospital (n = 499).	Hospital group: oral amoxicillin (50 mg/kg/day) administered for first 48 hr in the hospital followed by 5 days at home. Home group: 7 days oral treatment with amoxicillin (50 mg/kg/day) at home.	Primary outcome: treatment failure up to or on day 6. Secondary outcome: treatment failure between day 6 or and day 14; relapse.	Any of the following: clinical deterioration after enrolment, change of antibiotic, hospitalization, serious adverse event related to amoxicillin, LAMA, voluntary withdrawal of consent from study from enrolment by day 7, loss to follow up on day 8.	The treatment failures due to clinical deterioration in the home group –5.4% and in the hospital group –7.4%. Microbiological diagnosis was done. Two children died within the first 72 hrs, one in each group. There were no other serious adverse events. Neither of the deaths was considered to be related to oral amoxicillin.
Soofi et al [10] (cluster randomized)	Matiari district in Pakistan, community based involving lady health workers. Recruitment period: February 2008 to March 2010	Children aged 2–59 months with WHO-defined severe pneumonia. Oral amoxicillin (n = 2341) or co-trimoxazole (n = 2069) followed by referral.	**Intervention clusters:** oral amoxicillin for 5 days (90 mg/kg/day).**Control clusters:** first dose of oral co-trimoxazole (2–11 months, SMZ 200 mg plus TMP 40 mg; 12 months - 5 yrs, SMZ 300 mg plus TMP 60 mg) and then referred.	**Primary outcome:** treatment failure up to or on day 6.**Secondary outcome:** treatment failure between days 7–14; relapse.	Any of the following: appearance of any signs of very severe pneumonia, change of antibiotic treatment without objective criteria of treatment failure, persistence of fever >38°C with LCI on day 3 (after 48 h of initiation of treatment), either fever >1000F or LCI alone at day 6, death.	Treatment failure in intervention and control cluster was, 8% and 13% by day 6. Microbiological diagnosis was not done. Recorded 3 deaths, by day 6 (2 in the intervention group), and by day 9 (1in the control group).

**Note:** LCI = lower chest indrawing; LAMA = left against medical advice; SMZ = sulfa-methoxazole; TMP = trimethoprim; BD = twice daily.

### Risk of Bias in Included Studies

Studies were assessed by following criteria for any risk of bias e.g., details of sequence generation, allocation, blinding, incomplete outcome data, selective reporting, and other potential sources of bias (support or funding, ethics clearance) (**Figure S1**). All the studies were found to be of good qualities.

### Effect of Interventions

**1. Primary outcome measures.**

*Oral amoxicillin versus injectable antibiotics*

**Figure 2:**
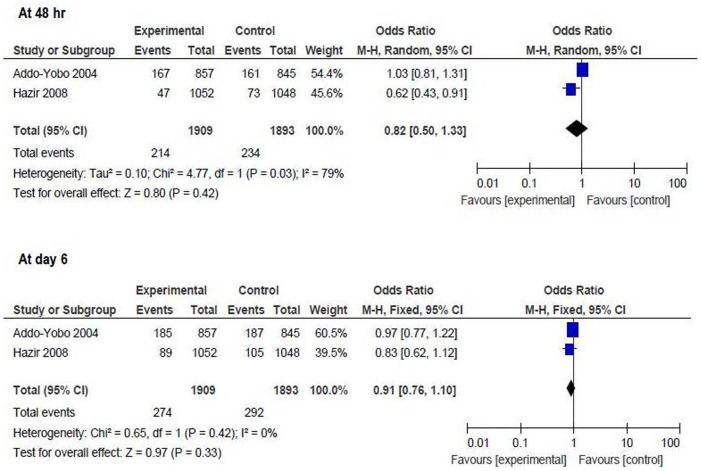
Primary outcome measures [oral amoxicillin versus injectable antibiotics]. Proportion of children developing treatment failure at 48 hr and at day 6.

- Proportion of children developing treatment failure at 48 hr: the result could be pooled from 2 studies including 3802 children (intervention = 1909, control = 1893) [6], [7]. There was no difference between the two groups [OR 0.82 (95% CI 0.5 to 1.33), p = 0.42] (**Figure 2**).
- Proportion of children developing treatment failure at day 6: the result could be pooled from 2 studies including 3802 children (intervention = 1909, control = 1893) [6], [7]. There was no difference between the two groups [OR 0.91 (95% CI 0.76 to 1.1), p = 0.33] (**Figure 2**).

*Oral amoxicillin versus oral cotrimoxazole and referral*

**Figure 3:**
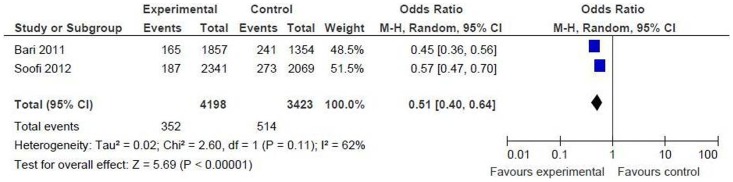
Primary outcome measures [oral amoxicillin versus oral cotrimoxazole and referral]. Proportion of children developing treatment failure at day 6.

Proportion of children developing treatment failure at day 6: the result could be pooled from 2 studies including 7621 children (intervention = 4198, control = 3423) [8], [10]. There was significant difference between the two groups [OR 0.51 (95% CI 0.40 to 0.64), p = <0.00001], suggesting that there is a 49% reduction in the risk of treatment failure in the amoxicillin group compared to oral cotrimoxazole and referral (**Figure 3**).

*Oral amoxicillin versus others (injectable antibiotics, oral cotrimoxazole and referral)*

**Figure 4:**
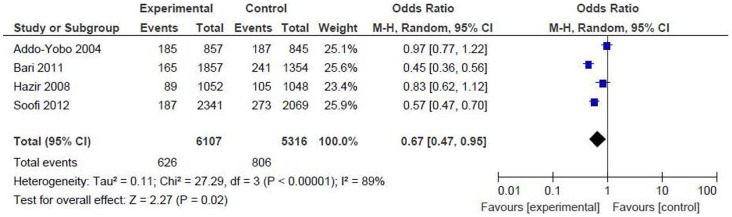
Primary outcome measures [oral amoxicillin versus others]. Proportion of children developing treatment failure at day 6.

Proportion of children developing treatment failure at day 6: we combined and pooled the result from above 4 studies including 11423 children (intervention = 6107, control = 5316) [6]–[8], [10]. There was significant difference between the two groups [OR 0.67 (95% CI 0.47 to 0.95), p = 0.02], suggesting that there is a 33% reduction in the risk of treatment failure in the amoxicillin group compared to others (**Figure 4**).

**2. Secondary outcome measures:**

*Oral amoxicillin versus injectable antibiotics*

**Figure 5:**
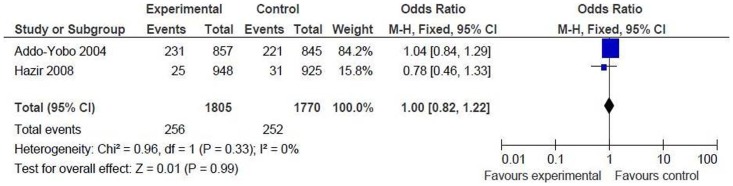
Secondary outcome measures [oral amoxicillin versus injectable antibiotics]. Proportions of children developing treatment failure or relapse by day 14.

- Proportion of children developing treatment failure or relapse by day 14: the result could be pooled from 2 studies including 3575 children (intervention = 1805, control = 1770) [6], [7]. There was no significant difference between the two groups [OR 1.0 (95% CI 0.82 to 1.22), p = 0.99] (**Figure 5**).

*Oral amoxicillin versus oral cotrimoxazole and referral*

**Figure 6:**
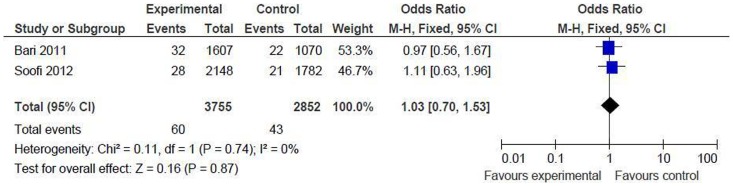
Secondary outcome measures [oral amoxicillin versus oral cotrimoxazole and referral]. Proportions of children developing treatment failure or relapse between day 6 and 14.

- Proportion of children developing treatment failure or relapse between day 6 and 14: the result could be pooled from 2 studies including 6607 children (intervention = 3755, control = 2852) [8], [10]. There was no significant difference between the two groups [OR 1.03 (95% CI 0.70 to 1.53), p = 0.87] (**Figure 6**). This is in contrast to the significant treatment failure rate at day 6, and can be explained by poor compliance. Most of the children in the oral cotrimoxazole group did not comply with referral and continued the antibiotic at home leading to increased treatment failure rate.

*Oral amoxicillin versus others (injectable antibiotics, oral cotrimoxazole and referral)*

**Figure 7:**
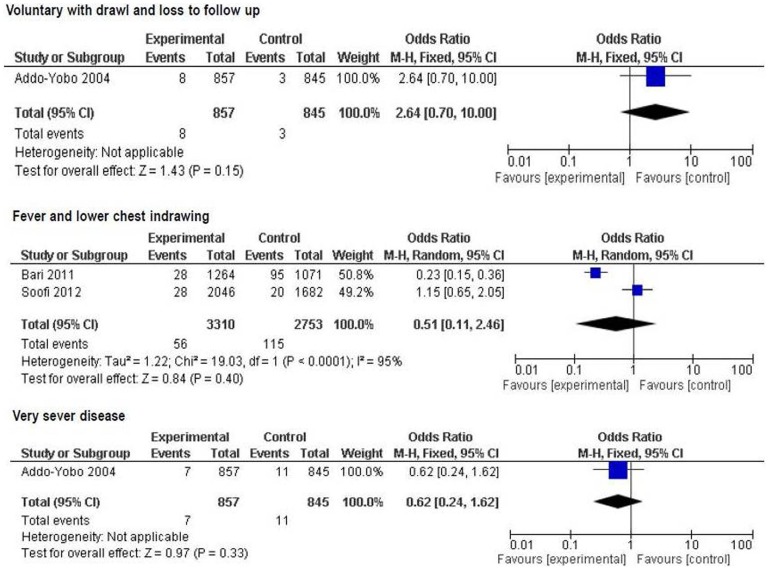
Secondary outcome measures: clinical predictors of treatment failure by 48 hr.

Various clinical predictors for treatment failure at 48 hr and at day 6 were analyzed. By 48 hr, none of the factors reported was a significant predictor of treatment failure between the groups (**Figure 7**).

**Figure 8:**
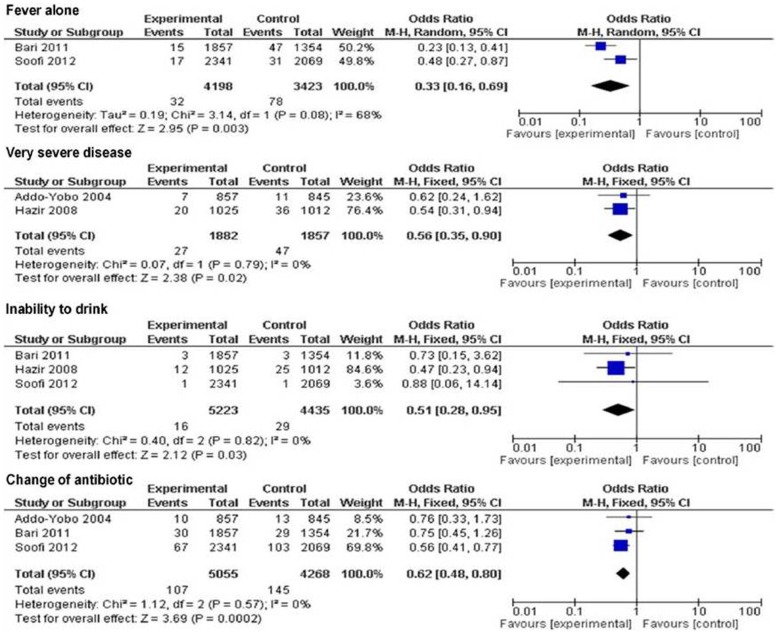
Secondary outcome measures: clinical predictors of treatment failure by day 6.

By day 6, fever alone [2 trials, OR 0.33 (95% CI 0.16 to 0.69), p = 0.003], very severe disease [2 trials, OR 0.56 (95% CI 0.35 to 0.90), p = 0.02], inability to drink [3 trials, OR 0.51 (95% CI 0.28 to 0.95), p = 0.03], and change of antibiotic [3 trials, OR 0.62 (95% CI 0.48 to 0.80), p = 0.0002] were found to be significant predictors of treatment failure between the groups (**Figure 8**).

#### Risk of Death

Though statistically not significant, the risk of death was less in oral amoxicillin group than in the other groups both at 48 hrs [1 trial, OR 0.07 (95% CI 0.00 to 1.14), p = 0.06] [6], and day 6 [3 trials, OR 0.4 (95% CI 0.12 to 1.31), p = 0.13] [6], [8], [10].

#### Grade of Evidence

For assessment of the quality of evidence we used GRADE Profiler software (version 3.2) [16]. The software uses five parameters for rating the quality of evidence. The parameters used are - limitations to design of randomized controlled trials, inconsistency of results or unexplained heterogeneity, indirectness of evidence, imprecision of results, and publication bias. The rating is done as – no, serious, and very serious limitation. The evidence generated for oral amoxicillin versus injectable antibiotics was of “very low quality” and versus oral cotrimoxazole plus referral was of “low quality” (**Table S1 and Table S2**).

#### Oral Amoxicillin Administered at Home Versus at Hospital

Only one trial [9] including 1118 children (intervention = 554, control = 564) studied the efficacy and safety of oral amoxicillin administered at home versus at hospital. The proportion of children developing treatment failure at day 7 was 8.5% in home versus 10.5% in hospital group [risk difference −1.5% (95% CI −5.1 to 2.1), p = 0.4). There was no difference in adjusted odds ratio of cumulative failure at ≤7 days between the two groups. The same trial also examined the proportion of children developing treatment failure or relapse between day 8 and 14, but found no significant difference between the two groups [OR 1.23 (95% CI 0.53 to 2.86), p = 0.64].

### Discussion

In the present meta-analysis we compared oral amoxicillin (administered either in hospital or home) versus either injectable antibiotics followed by oral amoxicillin at home or first dose of cotrimoxazole and then referral. We found that oral amoxicillin is as effective as the standard treatment of WHO defined severe CAP in children under-five. There was also no difference whether amoxicillin was administered at home versus at hospital. None of the clinical predictors of treatment failure by 48 hr (very severe disease, fever and lower chest indrawing, and voluntary with-drawl and loss to follow up) was significant between the two groups. The clinical predictors of treatment failure that were significant by day 6 were very severe disease, inability to drink, change of antibiotic, and fever alone. There was no difference in the treatment failure rate when we separately analyzed the data between the two treatment groups for infants of 2–11 months age [4 trials, OR 0.92 (95% CI 0.8 to 1.06), p = 0.25] [6]–[8], [10] and children of 12–59 months age [4 trials, OR 1.05 (95% CI 0.89 to 1.24), p = 0.58] [6]–[8], [10]. As mentioned above, though statistically not significant, the risk of death was less in oral amoxicillin group than in the other groups both at 48 hrs and at day 6.

In the management algorithm of severe pneumonia or very severe disease, WHO recommends that health providers administer the first dose of the appropriate antibiotic and then refer the child to nearest health facility for hospitalization and for administration of parenteral antibiotics along with other supportive therapy [17]. But in a developing country setting, accessing a referral facility is both logistically and financially difficult for the parents, which results in deprivation of these children from getting appropriate care. As a result, many children with severe pneumonia or very severe disease who are referred for treatment in a health facility either die on the way or got so sick at arrival at the health facility that nothing more can be done to save them [18] Besides these, children with severe pneumonia or very severe disease are vulnerable to superadded infections secondary to depressed immunity and are at increased risk of the same in crowded hospital wards, a common scenario in most of the developing or low-income countries.

The results of individual trials as well the present meta-analysis showed that, oral amoxicillin administered either in a hospital or community setting is equally efficacious as the injectable antibiotics and the same administered for the first 48 hrs in a hospital setting. This would have enormous implications for the health system as well as for the families that include - reduced costs of treatment for the families or community, improved care resulting from increased antibiotic coverage of severe pneumonia at the community level, decreased risk of hospital acquired infection or needle-associated complications, and reduced demand on the scarce inpatient services. It was observed in all the trials that, parents accept home-based treatment for severe pneumonia, which might be due to one or more reasons stated above.

The success and failure rates of treatment of community acquired pneumonia depend not only on the type of antibiotics but also on the etiology, age of the patient, sensitivity pattern of the bacterial pathogen, the severity of disease, any antibiotic usage in the recent past, and nutritional status of the child. Only two trials [6], [9] reported about the bacteriological diagnosis, but data on the resistance pattern was available in one study only [6]. In the multicentre trial (conducted in Africa, Asia, and South America), the rate of isolation of Streptococcus pneumoniae and Haemohilus influenzae was 28% and 20% respectively [6], where as in the trial from India, the rate of isolation of Streptococcus pneumoniae and Haemohilus influenzae was 18% and 9.7% respectively [9]. Therefore, empirical antibiotic therapy for community acquired pneumonia should be effective against these two pathogens. Intermediate-grade to high-grade resistance of S. pneumoniae to penicillin (67%) and low-grade resistance of Haemohilus influenzae to ampicillin (27%) was noted in the multicentre trial that reported about the resistance pattern [6]. The resistance pattern about amoxicillin was not reported in any of the trials. This is likely to be of major importance in the future in terms of both clinical practice and research, once amoxicillin is recommended as a standard initial treatment of severe pneumonia in community setting. Regarding the age groups mentioned above, there was no significant difference between infants of 2–11 months and children of 12–59 months in treatment failure rates. Data on prior antibiotic use was available in three trials [6], [7], [9]. Antibiotic use in <48 hrs prior to enrollment was associated with an increased risk of treatment failure in two trials; in one trial [9], hazard ratio 1.79 (95% CI 1.06 to 3.02) (p = 0.03), and in another, OR 1.84 (95% CI 1.27 to 2.66) [6]. Malnutrition may also affect the treatment outcome of pneumonia. Only one trial that reported severe malnutrition did not find it as a risk for treatment failure, OR 0.93 (95% CI 0.66 to 1.31) [6].

#### Strengths and Weaknesses of Review

Our meta-analysis included good quality trials, and though all were open label trials, allocation concealment was adequate in all. But the evidence generated from the present meta-analysis was “very low” when oral amoxicillin was compared versus injectable antibiotics and “low” when compared with oral cotrimoxazole and referral (**Table 2, Table 3**). Besides being open label studies, other factors that can explain the poor quality evidence are as follows. First, regarding comparison of oral amoxicillin versus injectable antibiotics, the dose of oral amoxicillin used in two trials was totally different; one [6] used lower dose where as the other [7] used high dose. At higher dose (80–90 mg/kg/day) amoxicillin in addition covers resistant Streptococcus Pneumoniae. One trial [6] used injectable penicillin, whereas the other [7] used injectable ampicillin. These two drugs have different spectrums with Haemophilus influenza being covered by ampicillin but not penicillin. Second, regarding comparison of oral amoxicillin versus oral cotrimoxazole and referral, factors like heterogeneity and imprecision played role in downgrading the evidence to low. As there was not enough trials neither we could do subgroup analysis nor test for publication bias. We also could not assess the effect of oral amoxicillin in severe CAP associated with lobar consolidation identified on chest X-ray, as none of the trials included radiological criteria. As all the studies were conducted in areas with low HIV prevalence, our result cannot be applied to population with high HIV prevalence.

Our systematic review has important differences from the two Cochrane reviews that have compared oral versus parenteral treatment of severe pneumonia [13], [19]. In one of these reviews, only two studies were included (one having high risk of bias) and the results were not pooled [13]. In another review, the authors compared oral amoxicillin with parenteral treatment only after including 3 trials, and also no synthesis of evidence was done [19]. In contrast to these reviews, we included good quality studies for synthesizing the evidence. Moreover, we compared oral amoxicillin versus oral cotrimoxazole and referral, and oral amoxicillin administered at home versus as hospital.

#### Further Area of Research

Though all studies found that oral amoxicillin is a possible appropriate treatment for severe CAP in under-five children, not all the studies reported about microbiological diagnosis and antimicrobial resistance. The dose of amoxicillin also varied among the studies. These factors will be important once administration oral amoxicillin is put into the algorithm of severe CAP case management guidelines. The comparators used were also different in all the trials. Though a single trial compared the administration of oral amoxicillin at home versus in hospital setting, we need more trials to validate the positive findings obtained from this single trial. There are many new antimicrobials available for the management of severe CAP. There is a need for more studies, using similar methodologies and large numbers of patients, to compare amoxicillin with co-amoxiclavulanic acid, macrolides with amoxicillin and amoxicillin with oral cephalosporins.

#### Conclusions

Though oral amoxicillin is a possible appropriate treatment for severe CAP in under-five children in developing country, the evidence supporting this is of low quality. So, we need more high quality trials with uniform comparators in order to strengthen the evidence that would help modifying the current WHO guideline for treatment of severe pneumonia in developing countries. Finding of a higher treatment failure rate with oral cotrimoxazole and referral also needs revisit of the current WHO guideline.

## Supporting Information

Figure S1 **Assessment of risk of bias in the included studies.**
(TIF)

Table S1 **GRADE Table.** Oral amoxicillin vs injectable antibiotics for severe pneumonia
(DOC)

Table S2 **GRADE Table.** Oral amoxicillin vs oral cotrimoxazole and referral for severe pneumonia
(DOC)
