# Supplementary figures and images for: Treatment of Severe Community-Acquired Pneumonia with Oral Amoxicillin in Under-Five Children in Developing Country: A Systematic Review
Source: PLoS One. 2013 Jun 25;8(6):e66232. doi: 10.1371/journal.pone.0066232 (PMC3692509; doi:10.1371/journal.pone.0066232)

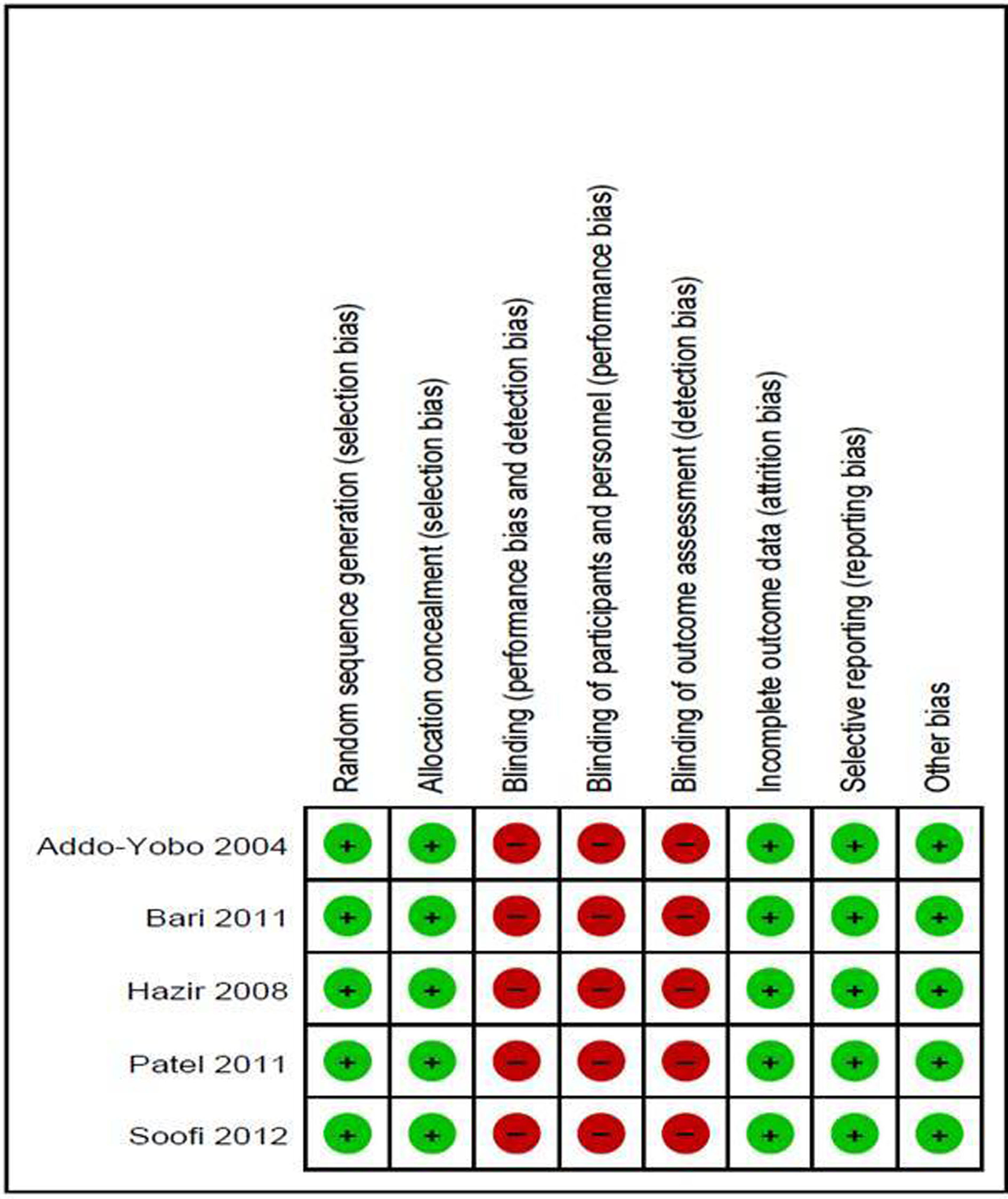

Supplement: Figure S1 — Assessment of risk of bias in the included studies. (TIF) [file pone.0066232.s001.tif]
