# Supplementary material for: Treatment of Severe Community-Acquired Pneumonia with Oral Amoxicillin in Under-Five Children in Developing Country: A Systematic Review
Source: PLoS One. 2013 Jun 25;8(6):e66232. doi: 10.1371/journal.pone.0066232 (PMC3692509; doi:10.1371/journal.pone.0066232)
Supplement: Table S1 — GRADE Table. Oral amoxicillin vs injectable antibiotics for severe pneumonia (DOC) [file pone.0066232.s002.doc]

**Table 2: GRADE Table. Oral amoxicillin vs injectable antibiotics for severe pneumonia**

| **Quality assessment** | | | | | | | **Summary of Findings** | | | |
| --- | --- | --- | --- | --- | --- | --- | --- | --- | --- | --- |
| Participants  (studies)  Follow up | Risk of bias | Inconsistency | Indirectness | Imprecision | Publication bias | Overall quality of evidence | Study event rates (%) | Relative effect (95% CI) | Anticipated absolute effects | |
|  |  |  |  |  |  |  | With injectable With oral  antibiotics amoxicillin |  | Risk with injectable Risk difference with antibiotics oral amoxicillin  (95% CI) | |
| ***Proportion of children developing treatment failure by 48hr*** | | | | | | | | | | |
| 3802  (2 studies) | serious | serious | serious | no serious imprecision | undetected | **VERY LOW**  due to risk of bias, inconsistency, indirectedness | 234/1893 214/1909  (12.4%) (11.2%) | **OR 0.82**  (0.5 to 1.33) | **Study population** | |
|  |  |  |  |  |  |  |  |  | **124 per 1000** | **20 fewer per 1000**  (from 58 fewer to 34 more) |
|  |  |  |  |  |  |  |  |  | **Moderate** | |
|  |  |  |  |  |  |  |  |  |  | _ |
| ***Proportion of children developing treatment failure by day 6*** | | | | | | | | | | |
| 3802  (2 studies) | serious | serious | serious | no serious imprecision | undetected | **VERY LOW**  due to risk of bias, inconsistency, indirectedness | 292/1893 274/1909  (15.4%) (14.4%) | **OR 0.91**  (0.76 to 1.1) | **Study population** | |
|  |  |  |  |  |  |  |  |  | **154 per 1000** | **12 fewer per 1000**  (from 33 fewer to 13 more) |
|  |  |  |  |  |  |  |  |  | **Moderate** | |
|  |  |  |  |  |  |  |  |  |  | _ |
| ***Proportion of children developing treatment failure or relapse by day 14*** | | | | | | | | | | |
| 3575  (2 studies) | serious | serious | serious | no serious imprecision | undetected | **VERY LOW**  due to risk of bias, inconsistency, indirectedness | 252/1770 256/1805  (14.2%) (14.2%) | **OR 1**  (0.82 to 1.22) | **Study population** | |
|  |  |  |  |  |  |  |  |  | **154 per 1000** | **0 fewer per 1000**  (from 23 fewer to 26 more) |
|  |  |  |  |  |  |  |  |  | **Moderate** | |
|  |  |  |  |  |  |  |  |  |  | _ |
