# Supplementary material for: Treatment of Severe Community-Acquired Pneumonia with Oral Amoxicillin in Under-Five Children in Developing Country: A Systematic Review
Source: PLoS One. 2013 Jun 25;8(6):e66232. doi: 10.1371/journal.pone.0066232 (PMC3692509; doi:10.1371/journal.pone.0066232)
Supplement: Table S2 — GRADE Table. Oral amoxicillin vs oral cotrimoxazole and referral for severe pneumonia (DOC) [file pone.0066232.s003.doc]

**Table 3: GRADE Table. Oral amoxicillin vs oral cotrimoxazole and referral for severe pneumonia**

| **Quality assessment** | | | | | | | **Summary of Findings** | | | |
| --- | --- | --- | --- | --- | --- | --- | --- | --- | --- | --- |
| Participants  (studies)  Follow up | Risk of bias | Inconsistency | Indirectness | Imprecision | Publication bias | Overall quality of evidence | Study event rates (%) | Relative effect (95% CI) | Anticipated absolute effects | |
|  |  |  |  |  |  |  | With oral With oral  cotrimoxazole amoxicillin |  | Risk with oral Risk difference with cotrimoxazole oral amoxicillin  (95% CI) | |
| ***Proportion of children developing treatment failure by day 6*** | | | | | | | | | | |
| 7621  (2 studies) | serious | serious | no serious indirectness | no serious imprecision | undetected | **LOW**  due to risk of bias, inconsistency | 514/3423 352/4198  (15%) (8.4%) | **OR 0.51**  (0.4 to 0.64) | **Study population** | |
|  |  |  |  |  |  |  |  |  | **150 per 1000** | **67 fewer per 1000**  (from 49 fewer to 84 fewer) |
|  |  |  |  |  |  |  |  |  | **Moderate** | |
|  |  |  |  |  |  |  |  |  |  | _ |
| ***Proportion of children developing treatment failure or relapse between day 6 and 14*** | | | | | | | | | | |
| 3802  (2 studies) | serious | no serious inconsistency | no serious indirectness | serious | undetected | **LOW**  due to risk of bias, imprecision | 43/2852 60/3755  (1.5%) (1.6%) | **OR 1.03**  (0.7 to 1.53) | **Study population** | |
|  |  |  |  |  |  |  |  |  | **15 per 1000** | **0 more per 1000**  (from 4 fewer to 8 more) |
|  |  |  |  |  |  |  |  |  | **Moderate** | |
|  |  |  |  |  |  |  |  |  |  | _ |
